# Promoting the Quality of Life of Elderly during the COVID-19 Pandemic

**DOI:** 10.3390/ijerph18136813

**Published:** 2021-06-25

**Authors:** Chia-Jung Lee, Yen Hsu

**Affiliations:** The Graduate Institute of Design Science, Tatung University, Taipei 104, Taiwan; d10717011@ms.ttu.edu.tw

**Keywords:** COVID-19, senior learning center, quality of life, augmented reality, life and health

## Abstract

This study explored the technology learning model of the elderly in a senior learning center under the impact of the COVID-19 pandemic. Many senior learning centers were closed during the pandemic, and many of them adopted the mode of online education. However, problems such as decreased motivation and a lack of peer interaction still exist. To solve these problems, this study used the easy-to-implement calligraphy AR approach and E-book approach to conduct a quasi-experiment on the elderly of a calligraphy course offered by a senior learning center. The results show a higher learning motivation among the elderly who use calligraphy AR. The learning effectiveness and technology acceptance of the elderly in the E-book learning group were higher than those in the calligraphy AR group. The elderly mentioned that the E-book learning approach is more user-friendly. In general, in the face of the COVID-19 pandemic affecting the suspension of classes in senior centers, education through the development of technology has stimulated the growth of education in advanced learning centers. Through this kind of scientific and technological learning method, it will bring a whole new experience to the elderly. It can improve the stress relief methods, mental health, and quality of life of the elderly during the COVID-19 emergency shutdown, and provide a novel calligraphy technique learning experience for the elderly. Therefore, we believe that the calligraphy AR learning approach and the calligraphy E-book learning approach are practical and may promote quality of life and mental health of the elderly during the emergency closures due to COVID-19, providing elderly attendees with a novel calligraphy technology learning experience.

## 1. Introduction

The COVID-19 pandemic has adversely affected people’s health worldwide and has negatively impacted the economy and educational fields. As a measure to contain the pandemic, many countries have ordered the cessation of any activity in educational institutions, even senior learning centers [1,2,3]. More and more schools are indeed adopting distance learning technologies for education and teaching. Teachers use various platforms to carry out teaching activities, such as video conferencing, e-mail, and Massive Open Online Course (MOOC). Although the adverse effects of the pandemic have been reduced significantly through such teaching activities [4], nevertheless, the lack of an overall school atmosphere has led to a decline in the self-discipline of some students. The learning motivation of students is weaker than offline study in schools. The learning interest and enthusiasm of students have also been affected to a certain extent [5]. The method of online learning and evaluation of students’ learning effectiveness has also become a concern of many teachers [3,6]. On the other hand, the influence of technology in life has been greatly increased during the period of the pandemic, and the technological applications have accelerated the innovation of education methods. In particular, augmented reality (AR) and artificial intelligence (AI) have been widely used in teaching, evaluation, and personal learning applications [7]. Many elderly people are forced to stay at home due to COVID-19, and they are facing problems of lack of social support and deterioration of their physical and mental health [8], and even increased family conflicts [9]. Technology-assisted learning can provide older adults with diversified activities at home, which can significantly help to improve life during the pandemic. Online learning has been an international development trend, and in response to the pandemic, the rapid development of online learning has been accelerated [10]. Current studies have suggested that online learning resources can effectively improve the learning effectiveness of lagging students [11]. AI technology can be used to analyze students’ learning progress and their learning effectiveness, so that teachers can adjust their teaching approaches to help students at different learning levels. At the same time, students can also develop the ability to learn independently after class [12]. Senior learning centers face similar problems in teaching approaches [13]. As older adults are more likely to suffer from severe COVID-19 illness [14], senior learning centers have been closed due to the pandemic. As COVID-19 prevents the face-to-face meeting of seniors and teachers, learning institutions have developed alternative teaching methods for online classrooms [14,15]. The calligraphy course has been a popular course among elderly learners, as calligraphy can significantly enhance the cognition and attention of elderly learners and improve their cognitive activities [16]. Learning calligraphy induces inner peace and abundance in elderly learners, enhances their concentration, care, patience, and perseverance, as well as improves the quality of life and the health of the elderly [17,18]. Therefore, this study attempts to find an effective calligraphy teaching approach to help the elderly learners maintain their mental health during the COVID-19 pandemic.

Mobile learning has attracted many older adults and can also improve their learning efficiency and motivation [19,20]. The convenience and effectiveness of using smartphones for learning activities has attracted educators worldwide [21,22,23,24]. Studies using smartphone technology in teaching methods can bring new learning models and effective learning methods [25,26,27]. The interactive features of augmented reality (AR) make it particularly suitable for teaching and learning. AR provides a more vivid, amusing, and unique visual experience to students [28,29]. AR can be widely applied in daily life, including games, medical treatment, navigation, social interaction, and extensive education [20]. AR has effectively improved students’ interest and teaching effects, and more and more educators are applying to teach [20,30,31]. In the elderly learning centers, AR technology can significantly help during the COVID-19 pandemic, school closures, and can improve online education shortcomings.

Mobile device-assisted learning is prevalent. Studies have focused on mobile learning in young people’s formal and non-formal education environments, such as informal education K-12 English courses, natural sciences, and physics or chemistry courses. The existing reports on learning through mobile devices were mainly associated with younger people [32,33], and there are only a few related studies on their use in informal and lifelong learning of the elderly. Peer assessment helps cultivate students’ learning abilities, with research confirming that anonymous peer evaluation can improve learning effectiveness [34]. These are more precise interpretations of the mutual criticism of learning content and evaluation criteria and the ability to develop good self-reflection skills [35,36,37]. Due to the limited research evidence of using peer-reviewed mobile learning methods in advanced learning centers for the elderly, the primary purpose of this research was to evaluate the use of mobile learning methods in calligraphy courses through experiments in such advanced learning centers. Therefore, we studied the effectiveness of the peer assessment in the calligraphy AR learning approach and the E-book learning approach in the calligraphy curriculum of the advanced senior learning center.

To the best of our knowledge, this is the first study to explore the effects of peer assessment of mobile learning on the calligraphy AR on the elderly of a senior learning center. We hope that this study will be a reference for other courses at senior learning centers during COVID-19.

The research questions are as follows.

- In the peer assessment, does the calligraphy AR approach show better learning effectiveness than the E-book approach?
- In the peer assessment, does the calligraphy AR approach show better learning motivation than the E-book approach?
- In the peer assessment, does the calligraphy AR approach show better self-efficacy than the E-book approach?
- In the peer assessment, does the calligraphy AR approach show better learning satisfaction than the E-book approach?
- In the peer assessment, does the calligraphy AR approach show better technology acceptance than the E-book approach?
- In the peer assessment, does the calligraphy AR approach show a lower cognitive load than the E-book approach?

## 2. Methods

### 2.1. Participants

The participants of this study were 75 elderly people (55–75 years old) enrolled in two calligraphy classes in a senior learning center. The participants continued their learning on smartphones at home for three weeks when the classes were suspended due to the COVID-19 surge. All participants had prior experience using smartphones. The screen size of the smartphones they used is 5.5–6.5 inches.

### 2.2. Research Design and Intervention

This study implemented a three-round learning activity using a quasi-experimental design. All classes used the online peer assessment approach for the experimental group, and the calligraphy AR learning approach was randomly assigned. For the control group, the online peer assessment, called the E-book learning approach, was assigned. The experimental tool is a system selected by three experts that is most suitable for the elderly. All the elderly participants had used mobile phones in the past.

**Figure 1:**
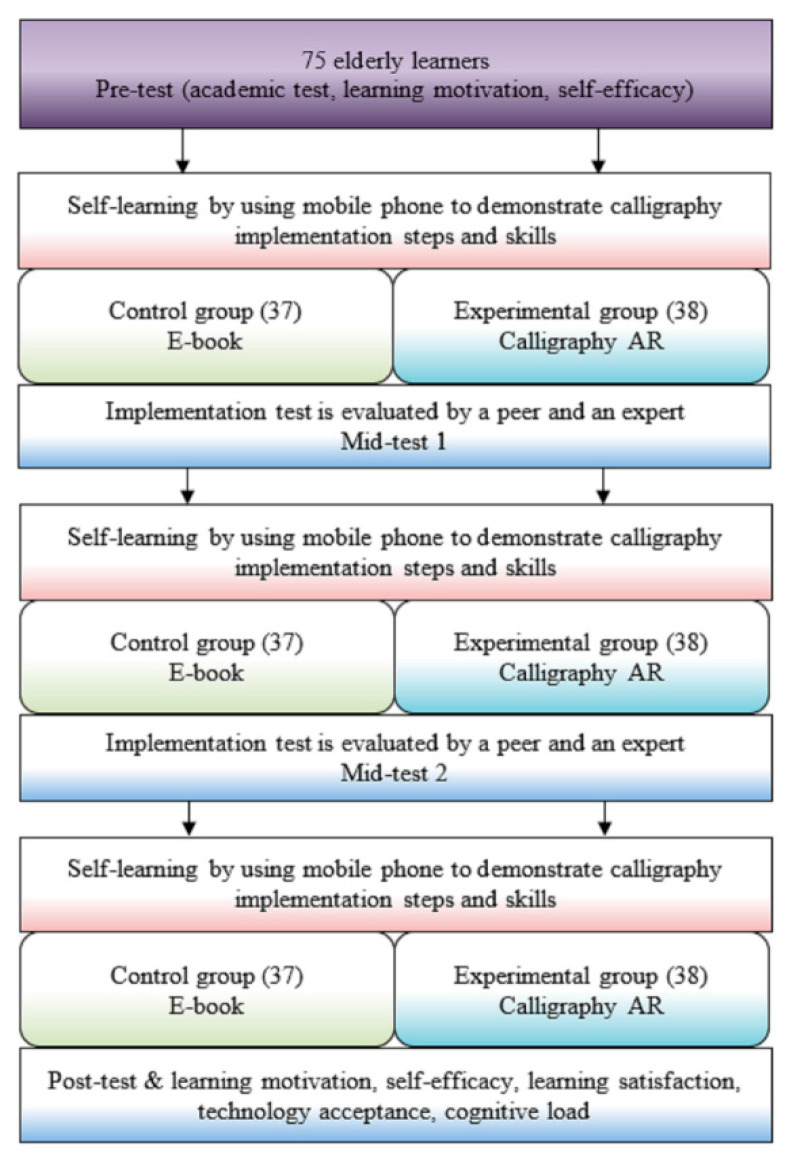
Experimental procedure.

Figure 1 compares the activity design of the experimental group and the control group. After each round of intervention, the elderly learners were asked to complete an online test concerning the calligraphy in a Chinese poem. After the third-round intervention, an online post-test was given to elderly learners in both groups.

**Figure 2:**
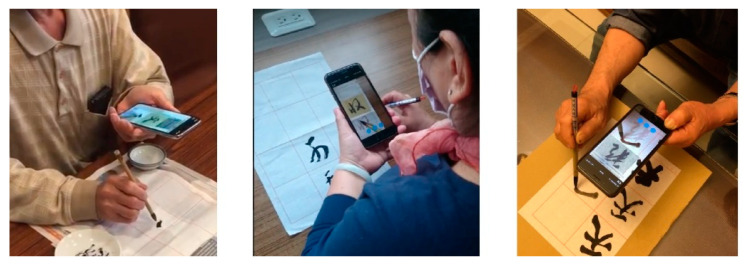
Schematics showing the operation of the calligraphy AR.

**Figure 3:**
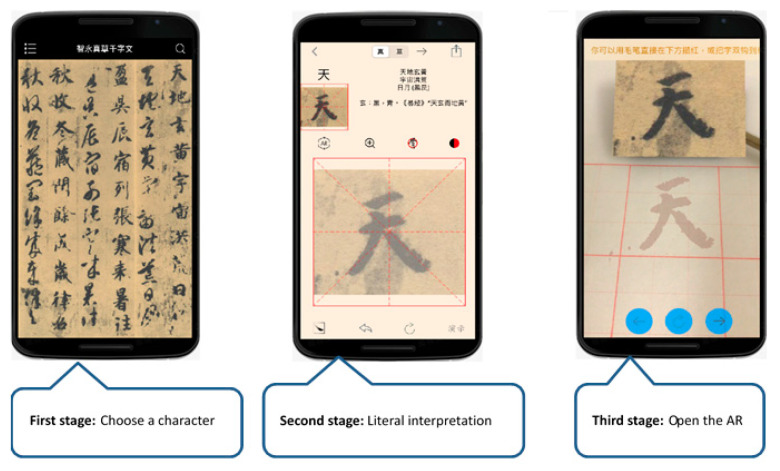
The experimental group of elderly participants using calligraphy AR.

**Figure 4:**
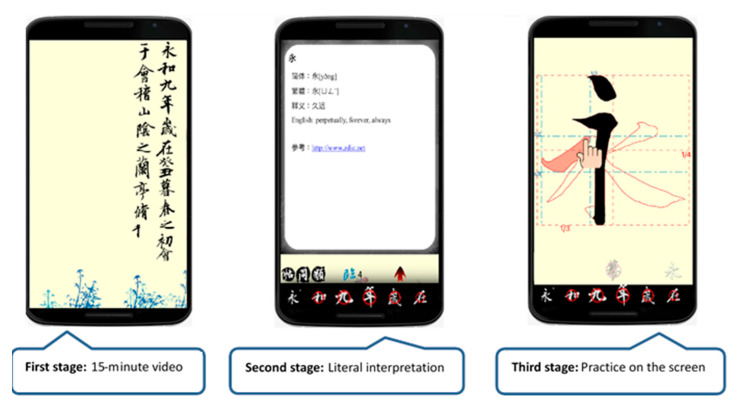
Schematics showing the operation of the calligraphy E-book.

**Figure 5:**
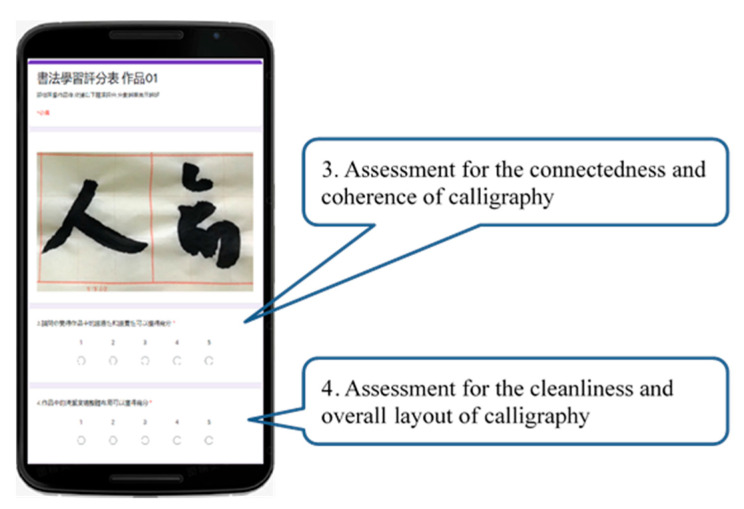
Screenshots of peer assessment items on participants’ mobile phones.

At the beginning of the experiment, the calligraphy AR and E-book approaches both explained the calligraphy contents of Chinese poems and illustrated the use of Chinese calligraphy brush strokes, alignment, spacing, connectedness, geometry, coherence, etc. The learners could choose the word they wanted to see. In the experimental group, the AR mode could detect a square shape and display the character in it, and display a transparent red image below for the user to write upon. Schematics showing the operation of the calligraphy AR, as shown in Figure 2. The experimental group of elderly participants using calligraphy AR, as shown in Figure 3. In the control group, the participants learned the content of E-books and watched an instruction video on calligraphy skills. The teaching content in the E-book included a video showing the steps of calligraphy, explanations, the meaning of the text, and a detailed introduction to the efforts of calligraphy Schematics showing the operation of the calligraphy E-book, as shown in Figure 4. The elderly learners in the control group practiced calligraphy on paper. After learning, all participants were asked to take photos of their calligraphy works and then evaluate each other’s works on a peer evaluation system using pre-designed rubrics. Screenshots of peer assessment items on participants’ mobile phones, as shown in Figure 5. The instructor used the same rubrics to evaluate the calligraphy works of the participants. After each session, both groups completed an online learning test.

### 2.3. Instrument Design

To measure the learning effectiveness, learning motivation, self-efficacy, learning satisfaction, technical acceptance, and cognitive load, tests were conducted before, during, and after the experiment. The pre-test mainly assessed whether the participants have equal knowledge on calligraphy before the experiment. The scale consisted of more than 50 multiple choice questions, with a maximum score of 100. During the teaching, two mid-term tests were conducted to assess the cognitive knowledge and calligraphy concepts of the elderly. The tests consisted of 30 multiple choice questions with 3 points for each question, and a short-answer question for 10 points, with a maximum score of 100 points. The tests were scored by two teachers with more than ten years of experience teaching calligraphy courses.

In addition to cognitive tests, the elderly were also required to perform hands-on exercises involving calligraphy. Peer evaluation rules were established for the hands-on calligraphy exercises by two experienced calligraphy teachers. The four main criteria are: (1) alignment, (2) spacing position, (3) connectivity and coherence, and (4) cleanliness and overall layout. Each criterion further includes five sub-criteria, which are scored between 1 (very poor) and 5 (very good), as shown in Figure 5.

The scale of learning motivation was modified from the test developed by Hwang and Chang [38], and it consisted of seven items (e.g., “I think learning calligraphy is interesting and valuable” and “I would like to learn more and observe more in the calligraphy course”) with a 5-point rating system. The Cronbach’s alpha value of the questionnaire was 0.79. The scale of self-efficacy was modified from the test developed by Wang and Hwang [39] and consisted of eight items with a 5-point Likert rating scale. The Cronbach’s alpha value of the questionnaire was 0.92. The questionnaire of a degree of satisfaction of using the learning approach was adopted from an earlier published study [40]. A total of seven items (e.g., “I like to learn with the learning approach” and “I would recommend this learning approach to others”) made up the questionnaire. The questionnaire in the original study stated a Cronbach’s alpha value of 0.89, implying good reliability in internal consistency. The scale of technology acceptance was modified from the questionnaire developed by Chu, Hwang, Tsai, and Tseng [40]. It consists of 13 items with a 5-point Likert scale, including 7 items for “Perceived ease of use” and 6 for “Perceived usefulness.” The Cronbach’s alpha values of the two dimensions were 0.94 and 0.95, respectively. The scale of cognitive load was modified from Hwang et al. [41] and consisted of two sizes with a total of eight items. The Cronbach’s alpha value of the questionnaire was 0.96.

## 3. Results

### 3.1. Analysis of Learning Effectiveness

**Figure 6:**
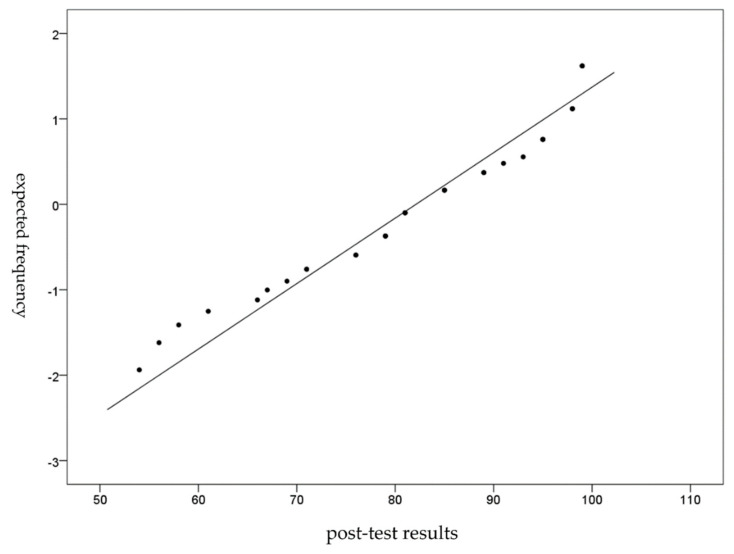
Post-test Q-Q plot.

Excluding the influence of the score before the questionnaire, the effectiveness of the participants’ learning outcomes after the learning activities was evaluated through the analysis of covariance (ANCOVA). The Shapiro–Wilk test was used to check the normality of the data. The value of this test was 0.97, *p* = 0.20, indicating that the research sample post-test results were normal distribution. The Q-Q plot (normal quantile-quantile plot) approximates a straight line to show that the sample is a standard distribution matrix, as shown in Figure 6.

Levene’s test of determining homogeneity of variance was not violated (*F* = 3.658, *p* > 0.05), indicating that the null hypothesis was tenable, and the conflict was equal across groups. In addition, the homogeneity of regression slopes was confirmed (*F* = 0.75, *p* > 0.05), suggesting that it was appropriate to employ the analysis of covariance. Thus, a one-way ANCOVA was conducted. The results of the ANCOVA indicated that the adjusted means and standard error were 83.41 and 0.60 for the experimental group and 85.17 and 0.59 for the control group respectively, as shown in Table 1. The two groups’ post-test scores were observed to be significant (*F* = 4.31, *p* < 05). Thus, there was a substantial difference in learning effectiveness between the elderly who used the calligraphy AR learning approach and those who learned through the E-book learning approach.

### 3.2. Analysis of Learning Motivation

The effectiveness of the participants’ learning motivation after the learning activity was evaluated through ANCOVA by excluding the impacts of the pre-questionnaire ratings. The Shapiro–Wilk value of this test was 0.98, and *p* = 0.27, indicating that the sample of this study had a normal distribution.

The test did not violate Levene’s test of determining homogeneity of variance (*F* = 0.42, *p* > 0.05), indicating that the null hypothesis was tenable, and the conflict was equal across groups. The homogeneity of regression slopes was confirmed (*F* = 1.102, *p* > 0.05), suggesting that it was appropriate to employ the analysis of covariance. Thus, a one-way ANCOVA was conducted. From the ANCOVA result, the adjusted means and standard error (SE) were respectively 3.57 and 0.08 for the experimental group and 3.30 and 0.08 for the control group, as shown in Table 2. Moreover, the post-test scores of the two groups were significantly different (*F* = 4.35, *p* < 05). In other words, there was a significant difference in learning effectiveness between the group who knew the calligraphy AR learning system and those who learned using the E-book learning approach.

### 3.3. Analysis of Self-Efficacy

The effectiveness of the participants’ self-efficacy after the learning activity was evaluated through ANCOVA by excluding the impacts of the pre-questionnaire ratings. The Shapiro–Wilk value of this test was 0.97, *p* = 0.26, indicating the normal distribution of the sample in this study.

Levene’s test for determining homogeneity of variance was not violated (*F* = 0.42, *p* > 0.05), indicating that the null hypothesis was tenable, and the conflict was equal among groups. In addition, the homogeneity of regression slopes was confirmed (*F* = 0.15, *p* > 0.05), suggesting that it was appropriate to employ the analysis of covariance. Therefore, a one-way ANCOVA was conducted. The ANCOVA results show that the adjusted means and standard error were 3.60 and 0.11 respectively for the experimental group and 3.31 and 0.11 respectively for the control group, as shown in Table 3. As seen, the post-test scores of the two groups were not different (*F* = 3.08, *p* > 0.05). In other words, there was no significant difference in self-efficacy between the group who learned from the calligraphy AR learning system and those who learned through the E-book learning approach.

### 3.4. Analysis of Learning Satisfaction

A *t*-test was employed to analyze the cognitive load of the elderly participants learning with the calligraphy AR learning system and the E-book learning approach to compare the results regarding their learning satisfaction. The results of the learning satisfaction *t*-test are shown in Table 4. The mean values and standard deviation (SD) of the post-questionnaire scores were respectively 3.65 and 1.05 for the E-book learning approach and 3.62 and 1.06 for the calligraphy AR learning approach, as shown in Table 4. There was no significant difference between the two groups. That is to say, the different learning systems did not result in differences in the elderly participants’ learning satisfaction after the learning activity.

### 3.5. Analysis of Technology Acceptance

A *t*-test was employed to analyze the technology acceptance of the elderly participants learning from the calligraphy AR learning system and through the E-book learning approach. The results of the technology acceptance *t*-test are shown in Table 5. The mean and standard deviation of the scores after the analysis of the perceived usefulness questionnaire were M = 3.37 and SD = 0.74 in the control group, and M = 3.14 and SD = 0.90 in the experimental group. The mean values and standard deviation of the perceived ease of use post-questionnaire scores were respectively M = 3.18 and SD = 1.14 for the control group and M = 2.32 and SD = 0.88 for the experimental group, as shown in Table 5. In other words, the elderly participants who learned with the E-book learning approach showed significantly better technology acceptance than those who learned from the calligraphy AR system. There were significant differences between the experimental group and the control group. That is to say, the elderly who used E-books to study calligraphy had better technology acceptance in terms of ease of use and usefulness.

### 3.6. Analysis of Cognitive Load

The cognitive load of the elderly participants was analyzed by the t-test, and the difference between the calligraphy AR learning approach and the E-book learning approach was compared. The results of the cognitive load *t*-test are shown in Table 6. The mean and standard deviation of mental load were M = 2.89 and SD = 1.01 in the experimental group and M = 2.39 and SD = 0.89 in the control group. The mean values and standard deviation of the perceived mental effort post-questionnaire scores were respectively 2.39 and 1.08 for the control group and 2.80 and 1.06 for the experimental group, as shown in Table 6. There was no significant difference between the two groups, i.e., the different learning systems did not differ in the participants’ cognitive load after the learning activity.

## 4. Discussion

The experimental results suggest that the E-book learning approach produced better learning effectiveness in the elderly than the calligraphy AR learning approach in the peer assessment. The calligraphy AR approach allowed the participants to obtain better learning motivation. The technology acceptance of the E-book learning approach, from the usefulness and ease of use perspective, was significantly higher than that of the calligraphy AR approach. As for self-efficacy, learning satisfaction, and cognitive load, there were no significant differences between the two groups.

This study confirmed that mobile learning has a very positive impact on the effectiveness of teaching calligraphy. First, in terms of learning achievements, the E-book approach resulted in better learning performances than the calligraphy AR approach did. This finding is different from previous studies that suggested that AR exhibited better learning performances. For the elderly, the E-book was easier to use, and the peer evaluation system was excellent in assessing elderly learners’ learning status. Therefore, for elderly learners, the E-book in mobile learning was practical.

Second, as for learning motivation, the results signified that the learning motivation of the elderly learners in the calligraphy AR group was better than those in the E-book learning group. The innovative use of calligraphy AR in the calligraphy course was novel and interesting for most elderly learners. Calligraphy AR could stimulate and improve the leaning motivation of elderly adults and learners. Especially for the online one-way knowledge dissemination during COVID-19, this novel calligraphy AR approach could undoubtedly bring new experiences and stimulation to elderly learners and provide more significant development potential for teaching. This finding is consistent with those of Chen, Chai, Jong, and Jiang [42,43] and Chen, Hung, and Yeh [42,43].

Third, in terms of technology acceptance, elderly learners in the E-book learning group exhibited better technology acceptance than those in the calligraphy AR group. Some older adults are reluctant to use new technologies, and only use mobile phones for communication or social functions. For most older adults, AR technology is considered unfamiliar [44]. On the other hand, due to the simple operation of the E-book, the technological acceptance of the elderly learners in the E-book group is higher than those in the calligraphy AR group. The elderly learners in the calligraphy AR group mentioned difficulties in using the calligraphy AR functions, while the elderly learners in the E-book group found the E-book easy to use, thus having a higher technological acceptance. According to the experimental results, the elderly learners had significant differences in the usefulness and ease of use aspect of technology acceptance. Through E-book learning, elderly learners’ acceptance of technology in both aspects significantly improved.

Finally, the experimental results did not show significant differences in self-efficacy, learning satisfaction, and cognitive load between the two groups. A possible explanation is that the short learning period did not improve the elderly participants’ self-efficacy and learning satisfaction. As Huang et al. and Yang et al. pointed out, elderly people need more time and learning experience to develop their abilities [31,45]. Moreover, the elderly participants in the experimental group were exposed to calligraphy AR for the first time, thus, three weeks of learning might not be long enough to improve their self-efficacy and learning satisfaction. It is common for older people to take a longer time to cultivate self-efficacy and learning satisfaction when using new technologies.

In addition, the calligraphy course is considered informal education, and the elderly learners might choose to spend more time on other social activities and interpersonal relationships to maintain their health and vitality [46]. For the elderly, learning calligraphy is a leisure activity and self-efficacy might not be their main focus in the course. The cognitive load was also not significant, which indicates that mobile learning is not difficult for the elderly. During the calligraphy course, there was no additional cognitive load when the elderly learners used the calligraphy AR mode. This finding is consistent with the results of Huang and Liaw [47]. Due to the temporary suspension of the senior learning center classes under the COVID-19 surge, face-to-face teaching is currently unavailable. Considering the effectiveness of online learning to improve the quality of life [48], interpersonal interaction could be achieved through the peer evaluation system for the course. This research used technology-assisted methods to allow the elderly participants to learn calligraphy safely at home, while achieving the effect of peer interaction. Under the continuing impact of the pandemic, technology-assisted learning of calligraphy can be regarded as an activity to promote health and improve the quality of life of elderly adults.

This study has the following limitations. First, the three-week teaching in this study was not long enough, and future research can extend the experimental period to verify the stability and sustainability of the research results of this study. Second, AR and E-book approaches can be applied to other courses offered by the senior learning centers to confirm their effects on different classes. At the same time, the sample size could be expanded to improve the accuracy of the experimental results. Finally, factors such as learning styles, gender, age, and educational level of the elderly could be included to expand the scope and depth of research.

## 5. Conclusions

This study focused on the effect of the technology-assisted learning of calligraphy for the elderly in the senior learning centers during COVID-19. As many elderly are forced to stay at home due to COVID-19, they face social support problems, and their physical and mental health deterioration has even been seen to increase family conflicts. Technology-assisted learning can provide older adults with diversified activities at home and can significantly help to improve quality of life during the pandemic. The results showed that the elderly have good results and motivation in using technology to learn. There was an excellent technological acceptance for usefulness and ease of use of the technology, and the convenience of system operation is essential for the elderly.

Moreover, novel and exciting AR technology can induce learning motivation in the elderly. We suggest that more AR or E-books suitable for 55–75-year-olds that are easy to operate be developed in the future, to ensure that the elderly who cannot go out can still enjoy leisure and entertainment at home. Senior learning centers were closed in the face of the COVID-19 pandemic, and education technology is a trend under development, which inspires growth in senior learning center education. This approach encourages the elderly to use it for stress relief and to maintain social interactions while at home in response to the pandemic, helping to maintain the mental health and quality of life of the elderly during COVID-19.

## Figures and Tables

**Table 1:** The ANCOVA result of learning effectiveness.

Group	N	M	SD	Adjusted Mean	SE	*F*	*η^2^*
Experimental Group	37	82.11	13.04	83.41	0.60	4.31 *	0.057
Control Group	38	86.45	12.25	85.17	0.59		

* *p* < 0.05.

**Table 2:** The ANCOVA analysis data of learning motivation.

Group	N	M	SD	Adjusted Mean	SE	*F*	*η^2^*
Experimental Group	37	3.50	0.80	3.57	0.08	4.35 *	0.057
Control Group	38	3.37	0.82	3.30	0.08		

* *p* < 0.05.

**Table 3:** The ANCOVA analysis data of self-efficacy.

Group	N	M	SD	Adjusted Mean	SE	*F*	*η^2^*
Experimental Group	37	3.54	0.96	3.60	0.11	3.08	0.041
Control Group	38	3.38	1.06	3.31	0.11		

**Table 4:** The independent *t*-test analysis of learning satisfaction.

Group	N	M	SD	SE	*F*	*t*
Experimental Group	37	3.62	1.06	0.17	0.13	−134
Control Group	38	3.65	1.05	0.17		

**Table 5:** The independent *t*-test analysis on technology acceptance.

Technology Acceptance	Group	N	M	SD	SE	*F*	*t*
Usefulness	Experimental Group	37	3.14	0.90	0.15	4.69 *	−1.224
	Control Group	38	3.37	0.74	0.12		
Ease of use	Experimental Group	37	2.32	0.88	0.14	5.56 *	−3.664
	Control Group	38	3.18	1.14	0.19		

* *p* < 0.05.

**Table 6:** The independent *t*-test analysis of cognitive load.

Cognitive Load	Group	N	M	SD	SE	*F*	*t*
Mental load	Experimental Group	37	2.89	1.01	0.17	0.19	2.286
	Control Group	38	2.39	0.89	0.14		
Mental effort	Experimental Group	37	2.80	1.06	0.17	0.29	1.683
	Control Group	38	2.39	1.08	0.18		

## Data Availability

The data presented in this study are available on request from the corresponding author. The data are not publicly available due to restrictions, e.g., privacy or ethical.
